# Real-world clinical features of and antidepressant prescribing patterns for outpatients with bipolar disorder

**DOI:** 10.1186/s12888-020-02967-5

**Published:** 2020-11-23

**Authors:** Keita Tokumitsu, Norio Yasui-Furukori, Naoto Adachi, Yukihisa Kubota, Yoichiro Watanabe, Kazuhira Miki, Takaharu Azekawa, Koji Edagawa, Eiichi Katsumoto, Seiji Hongo, Eiichiro Goto, Hitoshi Ueda, Masaki Kato, Reiji Yoshimura, Atsuo Nakagawa, Toshiaki Kikuchi, Takashi Tsuboi, Kazutaka Shimoda, Koichiro Watanabe

**Affiliations:** 1 Department of Psychiatry, Dokkyo Medical University, School of Medicine, Mibu, Shimotsuga, Tochigi 321-0293 Japan; 2 The Japanese Society of Clinical Neuropsychopharmacology, Tokyo, Japan; 3 The Japanese Association of Neuro-Psychiatric Clinics, Tokyo, Japan; 4 Department of Neuropsychiatry, Kansai Medical University, Osaka, Japan; 5 Department of Psychiatry, University of Occupational and Environmental Health, Fukuoka, Japan; 6 Department of Neuropsychiatry, Keio University School of Medicine, Tokyo, Japan; 7 Department of Neuropsychiatry, Kyorin University School of Medicine, Tokyo, Japan

**Keywords:** Bipolar disorder, Antidepressant, Nationwide study, Real world

## Abstract

**Background:**

Several evidence-based practice guidelines have been developed to better treat bipolar disorder. However, the articles cited in these guidelines were not sufficiently based on real-world clinical practice.

**Methods:**

The MUlticenter treatment SUrvey on BIpolar disorder in Japanese psychiatric clinics (MUSUBI) is a study conducted to accumulate evidence on the real-world practical treatment of bipolar disorder. Psychiatrists were asked to complete a questionnaire about patients with bipolar disorder by performing a retrospective medical record survey. The questionnaire included patient characteristics (age, gender, height, weight, academic background, and occupational status), comorbidities, mental status, treatment period, Global Assessment of Functioning (GAF) score, and details of pharmacological treatment.

**Results:**

Data on 2705 patients were included in this study. The proportion of patients receiving antidepressant prescriptions was 40.9%. The most commonly used antidepressant was duloxetine, and the most frequently used antidepressant class was selective serotonin reuptake inhibitors (SSRIs). Binomial logistic regression analysis and bivariate analysis revealed that the usage of antidepressants was correlated with low prescription rates for mood stabilizers, high prescription rates for anxiolytics and hypnotics, and low GAF scores. In addition, patients in a depressive state had a significantly higher rate of antidepressant prescriptions than patients with other mental states.

**Conclusions:**

Approximately 40% of patients in Japan with a diagnosis of bipolar disorder have received antidepressants. Antidepressants were most often prescribed in combination with mood stabilizers, antipsychotics or both. Patients who were prescribed antidepressants received fewer mood stabilizers, more anxiolytics, and more hypnotics than those who did not receive antidepressant prescriptions.

## Background

Bipolar disorder is an affective disorder that is characterized by repeated changes in activity or energy and is associated with characteristic cognitive, physical, and behavioral symptoms that impair the patient’s social functioning [1, 2]. The estimated lifetime prevalence of bipolar disorder among adults is 2.4% worldwide [3]. The average age at onset is 18 years for bipolar I and 20 years for bipolar II [3], and the prevalence in men and women is similar [4]. Problematic behavior caused by emotional changes often has negative social, financial, and occupational consequences [1]. The suicide risk of bipolar disorder patients has been reported to be 20 to 30 times higher than that of the general population [5]. Compared with manic and hypomanic episodes, bipolar depression and the symptoms of residual bipolar depression are associated with long-term morbidity and impaired function [6].

Several practice guidelines have been developed to better treat bipolar disorder. These evidence-based guidelines represent the current standard of treatment for bipolar disorder. However, the articles cited in these guidelines were clinical or basic studies conducted in specific conditions and settings and were not sufficiently based on real-world clinical practice. In recent years, the efficacy of antidepressants for patients with bipolar disorder has become an important issue. Guidelines for the treatment of bipolar disorder have been published by the Japanese Society of Mood Disorders (JSMD) and state that antidepressant monotherapy is not recommended for the mania phase or during rapid cycling [7]. However, they do not state that antidepressants should be avoided [7]. According to the National Institute for Health and Care Excellence (NICE) guidelines on bipolar disorder and the latest guidance, prescriptions of fluoxetine (a selective serotonin reuptake inhibitor (SSRI)) combined with olanzapine are recommended for moderate to severe depression [8]. On the other hand, the guidelines state that if a person develops mania or hypomania and is taking an antidepressant with or without a mood stabilizer, the cessation of antidepressants should be considered. Thus, in the treatment of bipolar disorder, antidepressant prescription is at the discretion of the physician [9].

Hence, research on prescriptions based on real-world evidence has attracted attention [10]. Research on real-world physicians’ prescribing behavior may identify medical suggestions that are missed by following current practice guidelines alone. A large-scale epidemiological study in Scotland found that approximately 60% of bipolar disorder patients were prescribed antidepressants [11]. It also revealed that the prescription rate of lithium carbonate, which is the first-choice drug for bipolar disorder [11], is only approximately 20% and is decreasing each year [11]. This trend was also consistent with the longitudinal data analysis results obtained from the National Ambulatory Medical Care Surveys [12]. In a study using Swedish national registries, 70% of patients with bipolar disorder were prescribed antidepressants [13]. In the United States, antidepressants are prescribed to 57.5% of patients with bipolar disorder [12]. In a study of nationwide and population-based prescription patterns in the Danish population, 59.12% of patients with bipolar disorder were prescribed antidepressants [14]. On the other hand, in a nationwide register-based study in South Korea, the antidepressant prescription rate for patients with bipolar disorder was 27.4–33.7% [15].

However, in Japan, the proportion of bipolar disorder patients who are prescribed antidepressants remains unclear. Additionally, there is a lack of large-scale clinical research on the actual prescription pattern of antidepressants for patients with bipolar disorder in Japan. Although practice guidelines are useful as a tool for achieving better prescription, real-world practice has limitations and can be wrong because of inappropriate prescribing habits [11]; therefore, a large-scale, multicenter observational study of the pharmacological treatment of patients with bipolar disorder is needed in Japan.

More than 90% of all patients with mood disorders in Japan are outpatients, half of whom are treated at clinics that are members of the Japanese Association of Neuro-Psychiatric Clinics. Against this background, joint research (the MUlticenter treatment SUrvey on BIpolar disorder in Japanese psychiatric clinics, or MUSUBI) was conducted between the Japanese Association of Neuro-Psychiatric Clinics and the Japanese Society of Clinical Neuropsychopharmacology to accumulate evidence on the real-world practical treatment of bipolar disorder in Japan. In this research, we assessed the rate of antidepressant prescription and its relation to clinical characteristics in outpatients with bipolar disorder in Japan.

## Methods

### Study design and subjects

MUSUBI is a cross-sectional study in which a questionnaire was administered at 176 outpatient clinics belonging to the Japanese Association of Neuro-Psychiatric Clinics from September to October 2016. We collected cross-sectional data on outpatients with bipolar disorder who visited each psychiatric clinic from October 1, 2016, to December 31, 2016. The analysis included only the current data for each patient at the time of the first visit during the study period (a single assessment time point). Each clinic collected data on up to 20 patients with bipolar disorder in the order that they visited the clinic [16]. Patients diagnosed with bipolar disorder based on the ICD-10 criteria [2] and treated at these clinics were included in this study. There were no exclusion criteria.

### Study procedures

Clinical psychiatrists were asked to complete a questionnaire about patients with bipolar disorder by performing a retrospective medical record survey. The questionnaire included patient characteristics (age, gender, height, weight, academic background, and occupational status), comorbidities, mental status, treatment period, Global Assessment of Functioning (GAF) score, and details of pharmacological treatment. The duration of prescriptions for various agents was not investigated. This study focused on antidepressant prescriptions for bipolar disorder patients and the profiles of these patients.

### Statistical analysis

All statistical analyses were performed with EZR (Saitama Medical Center, Jichi Medical University, Saitama, Japan) [17], which is a graphical user interface for R (The R Foundation for Statistical Computing, Vienna, Austria, version 3.5.2). More precisely, it is a modified version of R Commander (version 2.5–1) incorporating statistical functions that are frequently used in biostatistics.

All statistical tests were based on a two-sided significance level of 0.05. Demographic and clinical characteristics were analyzed using the chi-square test and the Mann-Whitney U test for differences between patients with and without antidepressant prescriptions. Univariate logistic regression analyses were performed to identify demographic and clinical features. All factors associated with antidepressant use among bipolar disorder patients were examined using binomial logistic regression with forced entry to avoid overlooking any potential associations. These factors included sex, body mass index (BMI), age at study entry, age at onset of bipolar symptoms, duration of attending clinics, current work status, educational level, mood stabilizer prescription (valproic acid, lithium carbonate, carbamazepine and lamotrigine), antipsychotic prescription, anxiolytic (benzodiazepines only) prescription, hypnotic prescription, intelligence quotient (IQ), GAF score, psychiatric comorbidity, personality disorder, developmental disorder, physical comorbidity, rapid cycling status, substance abuse, and mood status. The dummy variables included were as follows: male = 0, female = 1; employed = 1, unemployed = 0; some high school or high school graduate = 1, junior college = 2, college or postgraduate degree = 3; taking mood stabilizers =1, not taking mood stabilizers = 0; taking antipsychotics = 1, not taking antipsychotics = 0; taking anxiolytics = 1, not taking anxiolytics = 0; taking hypnotics = 1, not taking hypnotics = 0; IQ (> 85) = 0, IQ (85–71) = 1, IQ (< 71) = 2; GAF (81–100) = 0, GAF (61–80) = 1, GAF (41–60) = 2, GAF (< 41) = 3; psychiatric comorbidity = 1, no psychiatric comorbidity = 0; personality disorder = 1, no personality disorder = 0; developmental disorder = 1, no developmental disorder = 0; physical comorbidity = 1, no physical comorbidity = 0; rapid cycling = 1, no rapid cycling = 0; substance abuse = 1, no substance abuse = 0. Four mood states (depressive, mixed, remission and manic) were analyzed as nominal measures.

### Ethics

This study was conducted in accordance with the Declaration of Helsinki and the Japanese Ethical Guidelines for Medical and Health Research Involving Human Subjects. Prior to the initiation of the study, the study protocol was reviewed and approved by the institutional review board of the ethics committee of the Japanese Association of Neuro-Psychiatric Clinics. Since this was a retrospective medical record survey, it was exempted from the requirement for informed consent; however, we released information on this research so that patients were free to opt out [16]. The administrative permissions and licenses were acquired by our team to access the data used in our research.

## Results

Completed questionnaires on 3213 outpatients with bipolar disorder were returned from the 176 originally solicited outpatient facilities. A total of 508 outpatients with missing data were handled with listwise deletions. Thus, data on a total of 2705 patients were included in this study. The characteristics of the subjects are shown in Table 1.

**Table 1:** Demographic characteristics

Variables	Total (*N* = 2705)	Antidepressant (−) (*N* = 1598)	Antidepressant (+) (*N* = 1107)	*P* value	Scale type	Statistical method
Gender				0.240	nominal scale	chi-square test
Male: n(%)	1240 (45.8)	748 (46.8)	492 (44.4)			
Female: n(%)	1465 (54.2)	850 (53.2)	615 (55.6)			
Body mass index (kg/m2): mean	23.4	23.2	23.7	0.010	interval scale	Mann-Whitney U-test
Age at study entry: mean	50.2	50.7	49.6	0.074	interval scale	Mann-Whitney U-test
Age at onset of bipolar symptoms: mean	34.6	34.7	34.3	0.875	interval scale	Mann-Whitney U-test
Period of attending clinic (yaer): mean	7.9	7.6	8.3	0.004	interval scale	Mann-Whitney U-test
Current work status: n(%)				0.594	nominal scale	chi-square test
Employed	1808 (66.8)	1075 (67.3)	733 (66.2)			
Unemployed	897 (33.2)	523 (32.7)	374 (33.8)			
Educational level: n(%)				0.574	ordinal scale	Mann–Whitney U-test
Junior high school or high school graduate	1407 (52.0)	841	566			
Junior college graduate	240 (8.9)	135	105			
College or postgraduate degree	1058 (39.1)	622	436			
Mood stabilizer prescription: n (%)	2251 (83.2)	1395 (87.3)	856 (77.3)	< 0.001	nominal scale	chi-square test
Antipsychotics prescription: n (%)	1449 (53.6)	892 (55.8)	557 (50.3)	0.005	nominal scale	chi-square test
Anxiolytics prescription: n (%)	1000 (37.0)	472 (29.5)	528 (47.7)	< 0.001	nominal scale	chi-square test
Hypnotics prescription: n (%)	1605 (59.3)	896 (56.1)	709 (64.0)	< 0.001	nominal scale	chi-square test
Intelligence Quotient, IQ: n (%)				0.884	ordinal scale	Mann-Whitney U-test
> 85	2569 (95.0)	1517	1052			
85–71	106 (3.9)	60	46			
< 71	30 (1.1)	21	9			
GAF score: n(%)				< 0.001	ordinal scale	Mann-Whitney U-test
81–100	885 (32.7)	575 (36.0)	310 (28.0)			
61–80	1243 (46.0)	739 (46.2)	504 (45.5)			
41–60	512 (18.9)	252 (15.8)	260 (23.5)			
1–40	65 (2.4)	32 (2.0)	33 (3.0)			
Psychiatric comorbidity: n (%)	507 (18.7)	265 (16.6)	242 (21.9)	< 0.001	nominal scale	chi-square test
Personality disorder: n (%)	147 (5.4)	70 (4.4)	77 (7.0)	0.005	nominal scale	chi-square test
Developmental disorder: n (%)	177 (6.5)	97 (6.1)	80 (7.2)	0.264	nominal scale	chi-square test
Physical comorbidity: n (%)	821 (30.4)	468 (29.3)	353 (31.9)	0.160	nominal scale	chi-square test
Rapid cycler: n (%)	299 (11.1)	169 (10.6)	130 (11.7)	0.373	nominal scale	chi-square test
Substance abuse: n (%)	138 (5.1)	83 (5.2)	55 (5.0)	0.862	nominal scale	chi-square test
Mood status: n(%)				< 0.001	nominal scale	chi-square test
Depressive state	1098 (40.6)	519 (32.5)	579 (52.3)			
Manic state	230 (8.5)	186 (11.6)	44 (4.0)			
Mixed state	261 (9.6)	159 (9.9)	102 (9.2)			
Remission	1116 (41.3)	734 (45.9)	382 (34.5)			

*p* < 0.0025 was regarded as significant using Bonferroni’s correction due to multiple testing

*GAF* Global Assessment of Function

The proportion of patients who received antidepressant prescriptions was 40.9% (1107/2705). The combinations of antidepressant, mood stabilizer, and antipsychotic prescriptions are shown in Table 2. The most common combinations were a mood stabilizer and an antipsychotic, at 27.6%; mood stabilizer monotherapy, at 24.0%; a mood stabilizer and an antidepressant, at 16.6%; a mood stabilizer, an antipsychotic and an antidepressant, at 15.0%; an antipsychotic and an antidepressant, at 5.5%; antipsychotic monotherapy, at 5.4%; and antidepressant monotherapy, at 3.7%. A total of 2.1% of the patients were not prescribed a mood stabilizer, antipsychotic, or antidepressant.

**Table 2:** Patterns of combination among antidepressant, mood stabilizer, and antipsychotic prescriptions

	N	%
**With Antidepressant Use**	**1107**	**40.9**
Mood stabilizer and antidepressant	449	16.6
Mood stabilizer, antipsychotic and antidepressant	407	15.0
Antipsychotic and antidepressant	150	5.5
Antidepressant	101	3.7
**Without Antidepressant Use**	**1598**	**59.1**
Mood stabilizer and antipsychotic	747	27.6
Mood stabilizer	648	24.0
Antipsychotic	145	5.4
No prescription	58	2.1
**Total**	**2705**	**100.0**

The results of the univariate analysis are shown in Table 1. due to multiple (20) comparisons, a Bonferroni correction was applied, yielding a corrected significance criterion of *p* < 0.0025. By this threshold, the presence or absence of antidepressant use showed a statistically significant difference according to mood stabilizer prescriptions, anxiolytic prescriptions, hypnotic prescriptions, GAF scores, psychiatric comorbidities and mood status. Compared to the group that did not use antidepressants, the group using antidepressants had a lower rate of prescriptions for mood stabilizers (10%; *p* < 0.001) and higher rates of prescriptions for anxiolytics (18.2%; *p* < 0.001) and hypnotics (7.9%; *p* < 0.001). In addition, the group taking antidepressants had lower GAF scores (*p* < 0.001) and a higher proportion of psychiatric comorbidities (5.3%; *p* < 0.001) than the group not taking antidepressants. A higher proportion of patients were prescribed mood stabilizers only or mood stabilizers and antipsychotics (87.3%; 1395/1598) in the group not taking antidepressants than in the group taking antidepressants (77.3%; 856/1107). Therefore, prescription rates for mood stabilizers were relatively low in the antidepressant prescription group.

Regarding the antidepressant prescription rate associated with each mood state, 52.7% of prescriptions were associated with a depressive state, 39.1% with a mixed-features state, 34.2% with a remission state, and 19.1% with a manic state. A chi-square test between the 4 groups showed a statistically significant difference (*p* < 0.001). A four-group multiple comparison test showed that the depressive state group was prescribed antidepressants at a significantly higher rate than the other groups (*p* < 0.001). On the other hand, the proportion of antidepressant prescriptions was significantly lower for the manic group than for the other groups (*p* < 0.001). There was no statistically significant difference between the groups in the mixed state or the remission state (*p* = 0.959).

We calculated the mean GAF score for each mood status group using the class value. The GAF scores of the groups were as follows: depressive group, mean = 63.8; manic group, mean = 69.8; remission group, mean = 81.6; mixed-feature group, mean = 62.6. In addition, when a multiple comparison test using Tukey’s correction was performed, a statistically significant difference was observed between all pairs except for the depressive group and the mixed-features group.

A total of 20 antidepressants were prescribed for the patients with bipolar disorder (Table 3). The most commonly used antidepressant was duloxetine (a serotonin-norepinephrine reuptake inhibitor, or SNRI), and SSRIs were the most frequently used class of antidepressants. There were also some prescriptions, albeit relatively few, for tricyclic antidepressants (Table 3). A total of 848 patients were prescribed one antidepressant, 235 patients were prescribed two antidepressants, 23 patients were prescribed three antidepressants, and one patient was prescribed four antidepressants.

**Table 3:** Number of antidepressant prescriptions for patients with bipolar disorder

Antidepressants		Number and proportion of patients with each mood status									
Name	Class	Depressive (*n* = 1098)		Mixed features (*n* = 261)		Remission (*n* = 1116)		Manic (*n* = 230)		Total (*n* = 2705)	
Duloxetine	SNRI	138	13%	16	6%	57	5%	7	3%	218	8%
Mirtazapine	NaSSA	105	10%	11	4%	65	6%	7	3%	188	7%
Escitalopram	SSRI	101	9%	15	6%	37	3%	8	3%	161	6%
Trazodone	SARI	72	7%	16	6%	38	3%	7	3%	133	5%
Paroxetine	SSRI	54	5%	14	5%	49	4%	4	2%	121	4%
Fluvoxamine	SSRI	40	4%	9	3%	45	4%	7	3%	101	4%
Sertraline	SSRI	53	5%	10	4%	34	3%	3	1%	100	4%
Amoxapine	TCA	40	4%	8	3%	35	3%	1	0%	84	3%
Mianserin	TeCA	20	2%	6	2%	23	2%	3	1%	52	2%
Milnacipran	SNRI	25	2%	4	2%	22	2%	0	0%	51	2%
Others		101	9%	13	5%	62	6%	7	3%	183	7%

Binomial logistic regression analysis revealed that the patients with antidepressant prescriptions had a significantly higher BMI (odds ratio; OR [95% CI] = 1.04 [1.02–1.06], *p* < 0.001), longer duration of attending clinics (OR = 1.02 [1.01–1.04, *p* = 0.003]), lower prescription rate of mood stabilizers (OR = 0.48 [0.38–0.60], *p* < 0.001]), lower prescription rate of antipsychotics (OR = 0.58 [0.49–0.69], *p* < 0.001), higher prescription rate of anxiolytics (OR = 1.93 [1.63–2.28], *p* < 0.001), higher prescription rate of hypnotics (OR = 1.32 [1.11–1.57], *p* = 0.001), and lower GAF score (OR = 1.16 [1.01–1.33], *p* = 0.037) than those who were not prescribed antidepressants. Patients in the manic (OR = 0.22 [0.15–0.32], *p* < 0.001), mixed (OR = 0.57 [0.46–0.70], *p* < 0.001) and remission states (OR = 0.54 [0.40–0.73], *p* < 0.001) had significantly lower rates of antidepressant prescriptions than patients in the depressive state had. There was no significant difference between the proportions of rapid cyclers who did and did not take antidepressants (Table 4). In a binomial logistic regression analysis with forced entry, multicollinearity between variables was not observed.

**Table 4:** Factors associated with antidepressant prescriptions

Variables	Odds ratio	95% CI		*P* value
		Lower limits	Upper limits	
Gender	1.07	0.90	1.28	0.427
Body mass index	1.04	1.02	1.06	< 0.001
Age at study entry	0.99	0.98	1.00	0.007
Age at onset of bipolar symptoms	1.01	1.00	1.02	0.038
Period of attending clinic	1.02	1.01	1.04	< 0.001
Current work status	1.12	0.93	1.36	0.222
Educational level	1.05	0.96	1.15	0.281
Mood stabilizer prescription	0.47	0.38	0.58	< 0.001
Antipsychotics prescription	0.61	0.51	0.72	< 0.001
Anxiolytics prescription	1.98	1.68	2.34	< 0.001
Hypnotics prescription	1.34	1.13	1.58	< 0.001
Intelligence Quotient	0.83	0.61	1.12	0.225
GAF score	1.34	1.19	1.51	< 0.001
Psychiatric comorbidity	1.18	0.86	1.61	0.298
Personarity disorder	1.08	0.69	1.68	0.749
Developmental disorder	0.95	0.62	1.45	0.808
Physical comorbidity	1.10	0.92	1.32	0.306
Rapid cycling	1.07	0.82	1.39	0.618
Substance abuse	0.79	0.54	1.14	0.207

*p* < 0.05 was regarded as statistically significant using binomial logistic regression with forced entry

*GAF* Global Assessment of Function

We performed a multiple comparison test applying Tukey’s correction for the combinations of BMI and prescription type (antidepressants (AD), mood stabilizers (MS) and antipsychotics (AP)). Statistically significant differences were found according to BMI with the following combinations: MS + AP > no prescription (*p* = 0.010); AD+MS + AP > no prescription (*p* < 0.001); AD+MS + AP > MS monotherapy (*p* < 0.001); AD+MS + AP > AP monotherapy (*p* = 0.002); AD+MS + AP > AD+MS (*p* = 0.015).

## Discussion

Our MUSUBI baseline data analysis showed that antidepressants are prescribed for 40.9% of patients with bipolar disorder in a real-world clinical setting. Many of these patients receive antidepressants in combination with mood stabilizers and/or antipsychotics. Data from other countries have shown that antidepressant prescription rates for patients with bipolar disorder are approximately 30–70% [11–15, 18, 19], which is similar to the findings of our study. Especially in Korea, the prescription rate of antidepressants for patients with bipolar disorder is known to be increasing each year [15]. In a review comparing international practice guidelines, mood stabilizers or antipsychotics were recommended as the first-choice medication for bipolar depression. The efficacy and safety of long-term antidepressant use for bipolar II disorder has been emphasized in most guidelines [20].

According to the latest guidelines of the Canadian Network for Mood and Anxiety Treatments (CANMAT) and the International Society of Bipolar Disorders (ISBD) 2018 for the treatment of bipolar disorder [21], there is evidence based on a recent meta-analysis [22] that antidepressants are effective in treating acute bipolar depression and preventing depression symptoms in maintenance therapy. The guidelines also emphasized that antidepressant prescriptions should be considered clinically useful as a second-line add-on (combined with mood stabilizers) treatment for bipolar depression. The CANMAT and ISBD 2018 guidelines noted that historically, much of the focus has been on the risk of manic switching or rapid cycling, with an underappreciation of the relatively weak efficacy data [21]. This new appreciation for the efficacy of adjunctive antidepressant therapy led to a change in the CANMAT and ISBD 2018 guidelines, which also emphasized that this is a key aspect of decision making with regard to antidepressants.

Our study revealed that the most commonly used antidepressant for bipolar disorder outpatients was duloxetine, while the most frequently used antidepressant class was SSRIs. The latest CANMAT and ISBD 2018 guidelines [21] and the NICE guidelines [8] recognize the efficacy of adjuvant SSRI therapy, but there is insufficient evidence for the efficacy of duloxetine, which is an SNRI. This suggests that further prospective studies are needed regarding the efficacy of SNRIs in patients with bipolar disorder.

Regarding differences in demographic and clinical characteristics between patients with and without antidepressants, a previous study found that its antidepressant prescription group had a significantly higher proportion of women [11, 15]. In addition, it was reported that patients with antidepressant prescriptions had a high rate of conversion to a manic state [13] and a high comorbidity rate of anxiety disorders [15]. In particular, the prescription of antidepressants for bipolar disorder has been debated due to the risk of developing a manic state and the risk of increasing rapid cycling [13, 23–26]. However, in our study, there was no significant difference in the proportion of rapid cyclers between patients who took antidepressants and those who did not (*p* = 0.374). Moreover, in a comparison of antidepressant prescriptions between patients in a manic state and those in a remission state, the proportion of antidepressant prescriptions was significantly lower among the manic-state group (*p* < 0.001). Although additional treatment with antidepressants for bipolar depressive episodes did not improve mood in the short term compared to placebo [27], a recent meta-analysis reported that, in the long term, antidepressants have the potential to improve bipolar II depression in particular without increasing the risk of conversion to a manic state [28]. This meta-analysis also reported that the elevated risk of affective switching with antidepressant monotherapy compared with mood stabilizer monotherapy may contribute to the protective effect of mood stabilizers for diminishing manic/hypomanic episodes [28]. The results of this meta-analysis are considered to support the validity of prescribing behavior in the real world. On the other hand, since our study was a cross-sectional design, we cannot prove a causal relationship between antidepressant prescriptions and the risk of rapid cycling in bipolar disorder.

Regarding suicidality, there have been various reports that prescribing antidepressants in patients with bipolar disorder increases [29–31] or decreases the risk of suicide attempts [32, 33]. On the other hand, the rate of completed suicide in patients with bipolar disorder has been reported to have no significant difference between patients with and without antidepressant prescription according to logistic regression multivariant analysis [34]. Thus, there is contradictory evidence about the relationship between suicide and antidepressants. The CANMAT and ISBD 2018 guidelines do not provide a certain consensus on the causal relationship between antidepressant prescription and suicide in bipolar disorder due to limited data [21]. In any case, evaluation of treatment adherence is important. Because the common method of suicide in patients with bipolar disorder is self-poisoning, the potential benefits of various treatments should be considered against their risk of toxicity and lethality [21]. Since our study was a cross-sectional study, we could not assess the causal relationship between antidepressants and suicide in bipolar disorder.

Antidepressant monotherapy (without mood stabilizers and antipsychotics) is clearly associated with manic conversion and activation syndrome [13, 35–37]. However, it should be noted that 251 patients were prescribed antidepressants without mood stabilizers in the present study (9.3%; 251/2705), and 44 patients were prescribed antidepressants in the manic state (1.6%; 44/2705). Although antidepressant monotherapy was present (3.7%; 101/2705), the prevalence was lower than in Swedish [13] and Scottish [11] cohort studies. According to the latest evidence, patients with a history of antidepressant-induced mania or hypomania and recent rapid cyclers should avoid antidepressants, and antidepressant monotherapy should not be used to treat bipolar I depression [13, 37].

Unfortunately, some physicians in the clinic may have limited access to the latest information and may not be able to practice adequate evidence-based medicine; thus, there is the possibility that antidepressant monotherapy may be prescribed based on bad clinical practice. In addition, our logistic regression analysis revealed that the group that received antidepressants had a lower rate of mood stabilizer prescriptions. It is possible that bipolar depression may be treated as a major depressive disorder in the real world; alternatively, some patients who were misdiagnosed with bipolar disorder might have been included among our subjects.

Antidepressant prescriptions were associated with higher rates of anxiolytic and hypnotic prescriptions in the present study. This was consistent with the finding of the Bipolar CHOICE study that benzodiazepine users have high antidepressant prescription rates [38]. With regard to cognitive function in those with bipolar disorder, there are clear impairments associated with all mental states [39]. In particular, patients in a depressive state show greater impairment than euthymic patients on tests of verbal recall and fine motor skills [39]. Several reports have suggested that long-term use of benzodiazepine agents is associated with cognitive impairment [40, 41], but recently, some researchers have found that short-term use of benzodiazepine agents does not impair cognitive function [42]. Furthermore, they suggested that short-term use of benzodiazepine agents could improve information processing speed after acute treatment and at the 1-year follow-up [42]. However, in any case, there is a lack of evidence on the positive effects and adverse events of benzodiazepine for the treatment of bipolar disorder. The usefulness of concomitant benzodiazepine with antidepressants should be evaluated for each patient, and further longitudinal studies are required.

Interestingly, the BMI of patients receiving antidepressants was significantly greater than that of patients who were not prescribed antidepressants. In addition, the group taking antidepressants had significantly lower rates of antipsychotic and mood stabilizer prescription than the group not taking antidepressants. Thus, antidepressants may have a greater impact than antipsychotics on weight gain, although our study had a cross-sectional design and could not clarify the causal relationship.

In addition, binomial logistic regression analysis showed that the group of patients with antidepressant prescriptions had a significantly longer duration of attending clinic. It is known that patients with bipolar disorder have a longer duration of depression symptoms than manic symptoms [43]. A previous study revealed that the number of psychotropic agent prescriptions increased with prolonged treatment of bipolar disorder [44]. On the other hand, an unsuitable combination of antidepressant prescriptions may lead to polypharmacy and may adversely affect the patient’s mental state [45].

In our findings, the patients who were prescribed antidepressants had lower GAF scale scores. Because patients with bipolar depression are more likely to be prescribed antidepressants than patients in other phases, the GAF might have been administered during the bipolar depression phase [46]. A cross-sectional design was used in this study; thus, we could not determine a causal relationship between low GAF scores and prescriptions for antidepressants in our study population.

### Limitations

We did not distinguish between bipolar I and bipolar II and did not randomize patient selection; therefore, this study has limitations in terms of heterogeneity and selection bias in the patient population.

In addition, it was not possible to specifically distinguish between bipolar I and bipolar II with regard to the risk of mania during treatment and the potential protective effects of mood stabilizers or antipsychotics.

According to the guidelines, the appropriate antidepressant treatment for patients with bipolar disorder varies by subtype. Clinically, there is accumulating evidence that antidepressants are more effective in bipolar II than in bipolar I [21]. Additionally, it is emphasized that bipolar I should not be treated with antidepressant monotherapy [9]. However, this study does not distinguish between subtypes, which makes it somewhat difficult to determine which prescriptions pose a high risk in each subtype. This is one of the major limitations of our study. However, regardless of the subtype, antidepressants are effective against acute bipolar depression and have a certain degree of effectiveness in preventing depression during the maintenance phase [21]. On the other hand, the CANMAT and ISBD 2018 guidelines are not recommending antidepressant prescriptions for the manic state in any subtype of bipolar disorder [21].

Bipolar II patients may be prescribed antidepressants more frequently than bipolar I patients because of the deleterious consequences of antidepressants in the latter group. However, a longitudinal study that distinguished subtypes of bipolar disorder showed that both bipolar I and II had a much higher proportion of depressive symptoms (31.9 and 50.3%, respectively) and remission (52.7 and 46.1%, respectively) than cycling/mixed symptoms (5.9 and 2.3%, respectively) or manic/hypomanic symptoms (8.9 and 1.3%, respectively) [47, 48]. Our findings are largely consistent with previous studies. Therefore, although we were not able to distinguish bipolar subtypes, we can generalize the findings. Clinical evaluation in this study was performed by the doctors in charge of the patients; the doctors were well trained, but the diagnosis was not assessed through structural interviews. Thus, the possibility of some interrater variability cannot be excluded. Our study has a cross-sectional design, not a longitudinal design. Therefore, the causal relationships among the variables are unknown. Further longitudinal studies are needed to confirm the details of prescription patterns for patients with bipolar disorder in Japan.

Furthermore, in our study, we evaluated the manic-depressive mixed state, but we do not have accurate data on Mixed depression, defined as depression with excitatory symptoms [49]. Mixed depression may have been included in the depressive state or the mixed-feature state but could not be separated and extracted. Mixed depression is a fundamental topic in the evaluation and treatment of depression or bipolar disorder. Physicians should be aware that antidepressant prescriptions for mixed depression pose a high risk [49].

## Conclusions

Approximately 40% of patients with a diagnosis of bipolar disorder in Japan have received antidepressants. Antidepressants were most often prescribed in combination with mood stabilizers, antipsychotics or both. Patients who were prescribed antidepressants received fewer mood stabilizers, more anxiolytics, and more hypnotics than those who did not receive antidepressant prescriptions. Antidepressant prescription was correlated with higher BMI, lower GAF scores and patients in a bipolar depressive state.

## Data Availability

The datasets for the current study are available from the corresponding author on reasonable request.

## Abbreviations

MUSUBI: Multicenter treatment survey on bipolar disorder in Japanese psychiatric clinics
SSRI: Selective serotonin reuptake inhibitor
GAF: Global Assessment of Functioning
BMI: Body mass index
